# SCAN360: A Resource for a 360-Degree View of Cancer Prevention, Risk, and Survival

**DOI:** 10.5888/pcd17.200263

**Published:** 2020-11-25

**Authors:** Zinzi Bailey, Raymond Balise, Layla Bouzoubaa, Erin Kobetz

**Affiliations:** 1 Sylvester Comprehensive Cancer Center, University of Miami Miller School of Medicine, Miami, Florida; 2 Division of Medical Oncology, Department of Medicine, University of Miami Miller School of Medicine, Miami, Florida; 3 Division of Biostatistics, Department of Public Health Sciences, University of Miami, Miami, Florida

## Abstract

SCAN360, an interactive web platform aiming to provide a “360-degree view” of factors that drive cancer, calculates and integrates several measures of cancer burden from the Florida Cancer Data System, the state’s cancer registry, from 2012 to 2016 with cancer risk factors, clinical factors, and social determinants of health on multiple levels of geography — ranging from the entire state to the neighborhood. Integrating various sources of data, the web platform visualizes numerous indicators, including sociodemographic characteristics, cancer histology and staging, risk behaviors, screening behavior, environmental factors, hazardous sites, health insurance access, prevalence of potential comorbidities, housing characteristics, and levels of residential segregation, through maps and easy-to-interpret graphs. By walking through an example of a practical use, we show that SCAN360 provides data that are easily accessible to public health professionals, decision makers, and researchers and can assist them with identifying potential drivers of cancer burden on a localized level.

**Summary**

**What is already known on this topic?**

Understanding the patterns of cancer etiology, morbidity, and mortality across populations involves multiple levels of factors, ranging from the biological to the societal.

**What is added by this report?**

We describe the data sources, integration, calculations, and opportunities associated with SCAN360, an interactive web platform aiming to provide a “360-degree view” of factors that drive cancer burden in Florida, which can be replicated nationwide.

**What are the implications for public health practice?**

SCAN360 is an accessible resource for hospital administrators, community organizations, and public officials to make informed, comprehensive, and population-specific decisions.

## Background

As the second leading cause of death in the United States, cancer is a major public health problem in communities across the nation (1,2). Key behaviors (eg, smoking, heavy drinking, inactivity), infectious agents (eg, human papillomavirus [HPV], hepatitis B, hepatitis C, HIV, *Helicobacter pylori*), and genes (eg, *BRCA1*, *BRCA2*, *p53*) increase the risk of developing cancer (2–4). However, cancer is complex, and understanding its pattern across populations involves an interplay between multiple levels of factors, ranging from the biological to the societal. This understanding must include recognition of the role of physical and social environments in shaping lifestyles, habits, health-promoting resources, and prevalence of infectious agents (2). Although some states have developed systems that integrate area-level measures of social and built environments and geocoded cancer registry data (eg, California Neighborhoods Data System [2]), Florida did not have this kind of integrated system before the development of SCAN360, an interactive web platform aiming to provide a “360-degree view” of factors that drive cancer burden in Florida (5).

SCAN360 calculates and integrates several measures of cancer burden from the Florida Cancer Data System (FCDS), the state’s cancer registry, with cancer risk factors, clinical factors, and social determinants of health on multiple levels of geography — ranging from the state to the neighborhood. SCAN360 shows choropleth maps with color schemes that draw attention to areas with high values. The web platform visualizes various indicators, including sociodemographic characteristics, cancer histology and staging, risk behaviors, screening behavior, environmental factors, hazardous sites, health insurance access, prevalence of potential comorbidities, housing characteristics, and levels of residential segregation, through maps and easy-to-interpret graphs. SCAN360 is a tool used by cancer centers in their cancer control strategies, but it is also an accessible resource for hospital administrators, community organizations, and public officials to make informed, comprehensive, and population-specific decisions. This article describes the data sources, integration, calculations, and opportunities for research and public health practice associated with SCAN360, which can be replicated nationwide.

## Data Sources and Platform Development

Sponsored by the University of Miami’s Sylvester Comprehensive Cancer Center and the Miami Clinical and Translational Science Institute, SCAN360 provides tabular and graphical summaries, including interactive maps, for 19 types of cancer. It uses 4 measures: age-standardized 5-year incidence, mortality, prevalence of late-stage diagnosis, and the years of potential life lost attributable to cancer during 5 calendar years, 2012–2016. These statistics are available at many levels of aggregation, including 3 sex groups (male, female, everyone), 4 racial/ethnic groups (non-Hispanic White, non-Hispanic Black, Hispanic, all races), 4 age groups (youth [0–19 y], adult [20–64 y], senior [≥65 y], and everyone), and 5 levels of geography. The geographies — the entire state of Florida, all 67 Florida counties, 764 census-defined places (CDPs), and 11 Miami city districts — were selected to provide policy makers with meaningful locations.

Although cities, villages, and townships (what the Census calls CDPs) are critical to making policy decisions, the FCDS tags and reports cancer by census tract, not CDP. The US Census Bureau does not provide a direct mapping between census tracts and CDPs. Therefore, we used the MABLE GeoCorr 2014: Geographic Correspondence Engine, provided by the Missouri Census Data Center, to assign census tracts to CDPs (6). If a tract boundary crosses multiple CDPs, the cancer cases in that tract are assigned to the CDP with the largest population. This process avoids double counting, but it causes smaller places to be subsumed by neighboring, more populous CDPs. Therefore, SCAN360 reports on 764 of the 921 CDPs in Florida.

We standardized FCDS-provided data on incident cancer cases for 2012–2016 relative to the 2000 US Census. We calculated age-standardized mortality rates for the same by using data from the Florida Department of Health Bureau of Vital Statistics. We estimated both rates by using the direct standardization function in the *epitools* R package version 0.5–10.1 (R Foundation for Statistical Computing). We calculated years of potential life lost to cancer deaths by adapting the methodology for overall years of potential life lost to cancer deaths before age 65 instead of all deaths before age 65 (7). Because of restrictions imposed by the FCDS, the SCAN360 website does not display any cancer incidence or mortality rates for any geography that has fewer than 10 cases of cancer. We calculated other cancer statistics, such as the percentage of people diagnosed at a late stage (SEER [Surveillance, Epidemiology, and End Results] Summary stages 2 through 7) and the frequency of the histological variants (by cancer and race/ethnicity) by using functions from the *tidyverse* R package version 1.2.1 (8).

In addition to cancer statistics, SCAN360 offers summaries of relevant behavioral and environmental risk factors, including data on 18 unique factors known to influence cancer incidence and mortality, available through the American Community Survey (9). These factors include average commute time, access to a vehicle, insurance status, and 10 cancer risk and protective factors captured in the 2016 Behavioral Risk Factor Surveillance System (BRFSS), such as mammography and colorectal cancer screening. In choosing which risk and protective factors are the most relevant to the cancers included in SCAN360, the research team conducted literature reviews of sociodemographic, environmental, behavioral, clinical, and other risk factors, and sought advice from experts in the fields of cancer epidemiology, medical oncology, social epidemiology, and health disparities. We sourced data on environmental factors, such as the presence of toxins in the air, the amount of ultraviolet exposure, the air quality index, and the prevalence of food insecurity, from federal agencies such as the Environmental Protection Agency and the Centers for Disease Control and Prevention (Tables 1 and 2). This information is available on the SCAN360 website (scan360.com) (5).

**Table 1:** American Community Survey Tables^a^ and Associated Neighborhood Package Functions With Levels Used in SCAN360

Table Name	American Community Survey Table No.	Package Function	Levels
Sex by age	B01001	plot_age	5-year age groups by sex: 0–4/5–9/10–14/15–19/ . . . /75–79/80–84/≥85
Travel time to work	B08303	plot_commute	≥90/<90/<60/<45/<30/<15 min
Educational attainment	B15003	plot_education	Advanced degree/bachelor’s degree/ associate’s degree/some college/high school diploma or GED/some high school/<9th grade/no school
Language spoken at home	B16001	plot_english	Speak other than English/speak only English
Hispanic or Latino origin	B03003	plot_ethnicity	Hispanic or Latino/not Hispanic or Latino
Median household income in the past 12 months	B19013	plot_income	Actual median shown (no levels)
Health insurance coverage by sex and age	B27001	plot_insurance	By sex: male/female; by age: youth (0–17 y)/adult (18–64 y)/senior (≥65 y)
Health insurance coverage by sex, age, and type	B27012	plot_insurance_type	By sex: male/female; by age: youth (0–17 y)/adult (18–64 y)/senior(≥65 y); by type: employer-based/Medicaid/Medicare/private
Nativity	B05012	plot_native	Foreign-born/native
Percentage of population aged >65	B01001	plot_over65	Actual percentage shown (no levels)
Total population in occupied housing units by tenure	B25008_002/B25008_003^b^	plot_own_rent	Rent/own
Ratio of income to poverty level	C17002	plot_poverty	>2 times poverty threshold/<2 times poverty threshold/<poverty threshold
Race	B02001	plot_race	≥2 Races/Pacific Islander/Asian/Native American/Black/White
Gross rent as a percentage of household income	B25070	plot_rentincome	Extreme rent burden (>30%)/rent burden <30%
Employment status	B23025	plot_unemployment	Unemployed/employed
Vacancy status	B25004	plot_vacancy	Other vacant/for migrant workers/occasional use/sold, not occupied/for sale only/rented, not occupied/for rent
Means of transportation to work by vehicles available	B08141	plot_vehicle	≥1 Vehicle/no vehicle
Year structure built	B25034	plot_year_built	1939 or earlier/1940–1949/1950–1959/1960–1969/1970–1979/1980–1989/1990–1999/2000–2009/2010–2013/2014 or later

a US Census Bureau (9).

b Data for this factor were obtained from 2 separate variables from the same table.

**Table 2:** Indicators and Data Sources Used in SCAN360

Indicator	Source	Year	Type	URL
Superfund sites	Environmental Protection Agency	2018	Geocoded addresses	https://www.epa.gov/superfund/national-priorities-list-npl-sites-state#FL
Nuclear power plants	Nuclear Energy Institute	2018	Geocoded addresses	https://www.nei.org/CorporateSite/media/filefolder/resources/fact-sheets/state-fact-sheets/Florida-State-Fact-Sheet.pdf
Ultraviolet exposure	National Cancer Institute	2017	Rate	https://gis.cancer.gov/tools/uv-exposure
Radon exposure	Environmental Protection Agency	2017	Index	http://www.epa.gov/radon/zonemap.html; http://www.city-data.com/radon-zones/Florida/Florida.html
Limited access to healthy food	Robert Wood Johnson Foundation (RWJF) County Health Rankings; US Department of Agriculture (USDA) Food Environment Atlas	2015	Rate	http://www.countyhealthrankings.org/rankings/data/FL
Food insecurity	RWJF County Health Rankings; Map the Meal Gap from Feeding America	2015	Rate	http://www.countyhealthrankings.org/rankings/data/FL
Food environment index	RWJF County Health Rankings; USDA Food Environment Atlas; Map the Meal Gap from Feeding America	2015	Index	http://www.countyhealthrankings.org/rankings/data/FL
Exercise opportunities	RWJF County Health Rankings; Business Analyst; Delorme map data; ESRI; US Census Tigerline Files	2016	Rate	http://www.countyhealthrankings.org/rankings/data/FL
Percentage of population living within 0.5 mile of a park	Centers for Disease Control and Prevention (CDC) National Environmental Public Health Tracking Network	2011	Rates	https://ephtracking.cdc.gov/DataExplorer/#
Air quality index	Environmental Protection Agency	2018	Index	https://aqs.epa.gov/aqsweb/airdata/annual_aqi_by_county_2018.zip
Percentage of population within 150 m of highway	CDC National Environmental Public Health Tracking Network	2010	Rate	https://ephtracking.cdc.gov/DataExplorer/#
Percentage of public schools within 150 m of highway	CDC National Environmental Public Health Tracking Network	2010	Rate	https://ephtracking.cdc.gov/DataExplorer/#
Drinking water violations	RWJF County Health Rankings; Safe Drinking Water Information System	2016	Binary	http://www.countyhealthrankings.org/rankings/data/FL
Percentage of severe housing problems	RWJF County Health Rankings; Comprehensive Housing Affordability Strategy (CHAS) data	2010–2014	Rate	http://www.countyhealthrankings.org/rankings/data/FL
Residential segregation	RWJF County Health Rankings; American Community Survey, 5-year estimates	2012–2016	Index	http://www.countyhealthrankings.org/rankings/data/FL
Income inequality	RWJF County Health Rankings; American Community Survey, 5-year estimates	2012–2016	Ratio	http://www.countyhealthrankings.org/rankings/data/FL
Supplemental Nutrition Assistance Program (SNAP) redemptions/SNAP-authorized stores	USDA Food Environment Atlas	2018	Average dollar	https://www.ers.usda.gov/webdocs/DataFiles/48731/DataDownload.xls?v=0
Brownfields	Florida Geographic Data Library Metadata Explorer	2018	shp files	https://download.fgdl.org/pub/state/brownfields_areas_feb18.zip
Hazardous waste sites	Florida Geographic Data Library Metadata Explorer	2018	shp files	https://download.fgdl.org/pub/state/chaz_jul18.zip
Correctional facilities	Florida Geographic Data Library Metadata Explorer	2017	shp files	https://download.fgdl.org/pub/state/gc_correctionalbnd_sep17.zip
Fire stations	Florida Geographic Data Library Metadata Explorer	2018	shp files	https://download.fgdl.org/pub/state/gc_firestat_may18.zip
Health care facilities	Florida Geographic Data Library Metadata Explorer	2014	shp files	https://download.fgdl.org/pub/state/gc_health_aug14.zip
Parks	Florida Geographic Data Library Metadata Explorer	2017	shp files	https://download.fgdl.org/pub/state/gc_parks_sep17.zip
Schools	Florida Geographic Data Library Metadata Explorer	2017	shp files	https://download.fgdl.org/pub/state/gc_schools_sep17.zip
Mobile home and RV parks	Florida Geographic Data Library Metadata Explorer	2018	shp files	https://download.fgdl.org/pub/state/mhrv_mar18.zip
Mammography centers	US Food and Drug Administration	2017	Geocoded addresses	https://www.fda.gov/radiation-emittingproducts/mammographyqualitystandardsactandprogram/consumerinformation/ucm113962.htm
Special Supplemental Nutrition Program for Women, Infants, and Children centers	Special Supplemental Nutrition Program for Women, Infants, and Children	2018	Geocoded addresses	http://www.wicprograms.org/state/florida
Free clinics	FreeClinics.com	2018	Geocoded addresses	https://www.freeclinics.com/co/fl
HIV testing sites	Florida Department of Health	2018	Geocoded addresses	https://flhiv.doh.state.fl.us/ClinicSearch/textsearch.aspx?county=true
Pre-exposure prophylaxis (PrEP) sites	Florida Department of Health	2018	Geocoded addresses	https://flhiv.doh.state.fl.us/ClinicSearch/textsearch.aspx?area=true
Sylvester Comprehensive Cancer Center satellite sites	Sylvester Comprehensive Cancer Center	2018	Geocoded addresses	https://umiamihealth.org/sylvester-comprehensive-cancer-center/locations?category=all&currentaddress=&distance=10
Academic cancer centers	Florida Academic Cancer Center Alliance	2018	Geocoded addresses	http://floridacancerresearch.org
Department of Community Service health fairs	University of Miami Miller School of Medicine, Department of Community Service	2018	Geocoded addresses	https://umdocs.mededu.miami.edu/projects
Tobacco retailers	Florida Department of Business and Professional Regulation	2018	Geocoded addresses	https://www.myfloridalicense.com/wl11.asp?mode=1&SID=&brd=&typ=

We conducted all analyses for SCAN360 using R 3.5.3 software (R Foundation for Statistical Software). We extracted American Community Survey data for various geographies via API (application programming interface) calls using the *acs* R package version 2.1.4 (R Foundation for Statistical Computing); that is, we used the R programming language to communicate with the US Census Bureau without human intervention, and we calculated percentages for each level of geography using the base R and *tidyverse* functions. We defined Miami city districts by their census tracts. For each district, we calculated factor estimates by taking the average of that estimate weighted by the population in the census tract. Because BRFSS data are available only at the county level, we calculated rates of behavior (eg, mammography screening) for CDPs and districts from census tract–level data obtained from the 500 Cities Project (10).

SCAN360 enables the comparison of 2 rates for any cancer or contextual factor (behavioral/environmental/socioeconomic) for any combination of geography and population subgroup through side-by-side maps. We obtained the shapefiles used to draw the maps for the state, counties, and CDPs from the *tigris* R package version 0.8.2 (R Foundation for Statistical Computing). We created a shapefile for Miami city districts by grouping the census tracts that define them and spatially joining them using ArcMap version 10.6 software (Esri).

## Use of SCAN360 to Explore Cancer Incidence in Florida

**Figure 1:**
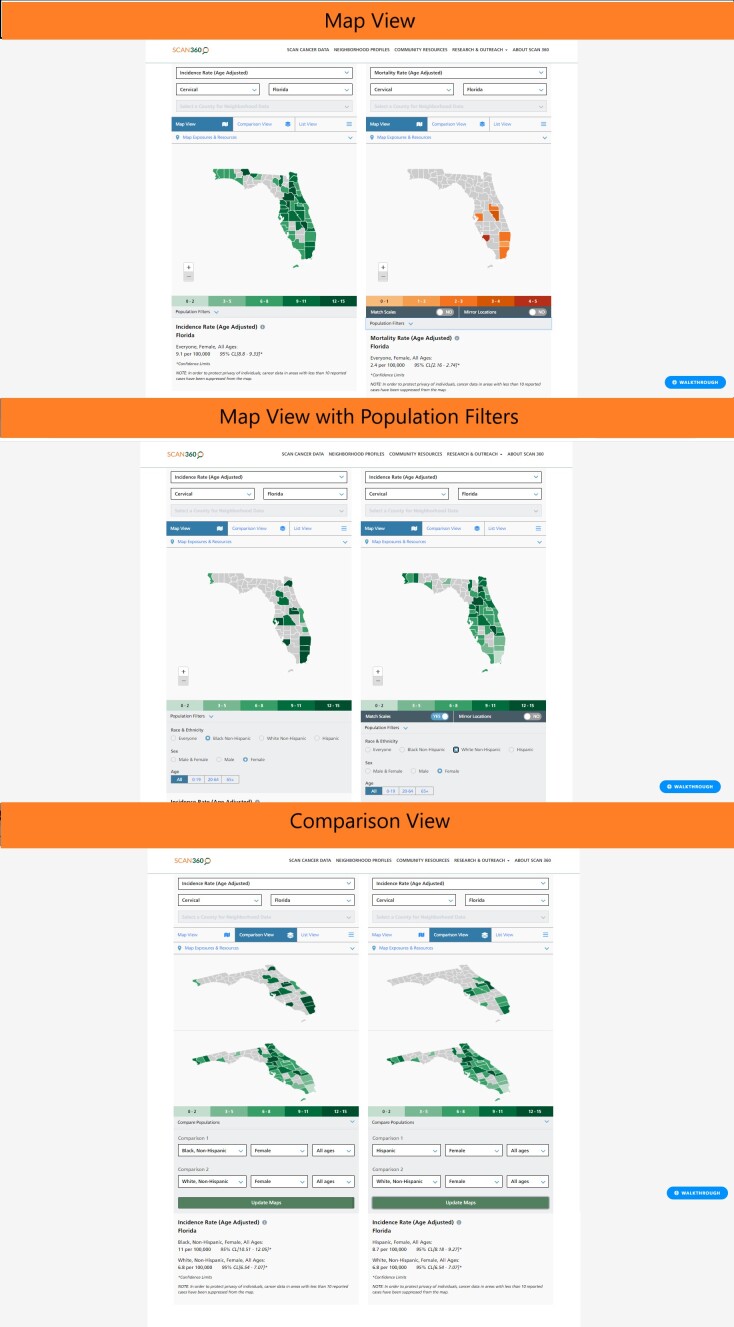
Map view, map view with population filters, and comparison view in SCAN360.

SCAN360 combines several measures of cancer burden into a single user-friendly platform (Figure 1). The higher rates in central and northern Florida in overall cancer incidence and mortality shown on the SCAN360 maps is consistent with rural health disparities and proximity to the Deep South (https://bit.ly/overallcancer) (11,12). However, when focusing on cancer control and prevention, it is important to recognize that some cancers disproportionately contribute to the cancer burden in some geographic areas. For example, in South Florida, the 4-county region of Miami-Dade, Broward, Palm Beach, and Monroe counties, is a sociodemographically heterogeneous area that accounts for nearly 30% of the population in Florida. Through SCAN360, this heterogeneity can be explored in relationship to drivers and correlates of cancer burden.

The incidence rates of cervical cancer in Miami-Dade, Broward, and Monroe counties stand out from neighboring counties (https://bit.ly/cervicalcancerFL). Furthermore, when exploring the population filters in SCAN360, cervical cancer in Miami-Dade County stands out at older ages, especially among women aged 65 or older, which can be seen in SCAN360 by using the hover feature over Miami-Dade County (https://bit.ly/agepopfilter). The estimated age-adjusted cervical cancer incidence rate among women aged 20 to 64 in Miami-Dade County is 14 per 100,000 (with a 95% CI that includes the statewide rate for the same age group), whereas the rate for women aged 65 or older in Miami-Dade County is 17 per 100,000 (vs 11 per 100,000 statewide in the same age group). The higher rate among older women likely reflects many factors, including geographic distribution of people across different ages, which can be investigated through further population filters. For example, we can also look at cervical cancer across race and ethnicity. When the population filters are used to focus on non-Hispanic Black women, Miami-Dade, Broward, and Palm Beach counties stand out among other counties in Florida as having the highest rates of cervical cancer (13 per 100,000; 14 per 100,000; and 13 per 100,000; respectively). To a lesser degree, we see the same counties in this catchment area stand out with high rates among Hispanic women (Miami-Dade, 9.3 per 100,000; Broward, 7.8 per 100,000; and Palm Beach, 6.6 per 100,000) compared to the rates in other counties (side-by-side maps available at https://bit.ly/SCANblackhispanic).

The high rates among non-Hispanic Black and Hispanic women in the catchment area are distinctive, especially when compared with the pattern of incidence among non-Hispanic White women in the same counties (https://bit.ly/SCANwhiteblack). Again, this racial/ethnic difference in the geographic distribution of cancer incidence is likely to reflect many factors, including the underlying distribution of racial/ethnic groups (with different age distributions) across different geographies, which can be more thoroughly investigated through further study. However, looking at the magnitude and precision of the disparity in incidence, we can infer that the incidence of cervical cancer in Miami-Dade, Broward, and Palm Beach counties is concentrated among Black (and to a lesser extent, Hispanic) women in South Florida.

We can look at these data in another way, through the comparison view, which magnifies the ability to display contrasts in incidence rates by geography and population (https://bit.ly/SCANCompView). Although contextualizing incidence rates in the broader landscape of Florida is helpful, documenting and visualizing disparities at the county level is not sufficient to guide targeted outreach and research. We must look more closely at the heterogeneity *within* each county. Zooming into Miami-Dade County in Map View, we get a sense of geographic heterogeneity (https://bit.ly/SCAN305). Choosing a county allows the user to select even smaller levels of geography — neighborhoods.

Zooming in to the neighborhood level, we see that neighborhoods such as Little Haiti, North Miami, Model City, West Little River, Golden Glades, Homestead, Leisure City, and University Park have the highest rates of cervical cancer in the county. Furthermore, if we restrict our investigation of cervical cancer incidence to non-Hispanic Black women, we observe the highest rates in Miami Gardens (16 per 100,000), Little Haiti (20 per 100,000), and North Miami (23 per 100,000) (https://bit.ly/SCANMIABH). If we restrict our investigation of cervical cancer incidence to Hispanic women, we see different neighborhoods stand out, including Hialeah, Allapatah, Little Havana, Miami Beach, and Homestead, not surprisingly predominantly Hispanic/Latinx communities (https://bit.ly/SCANMIABH).

**Figure 2:**
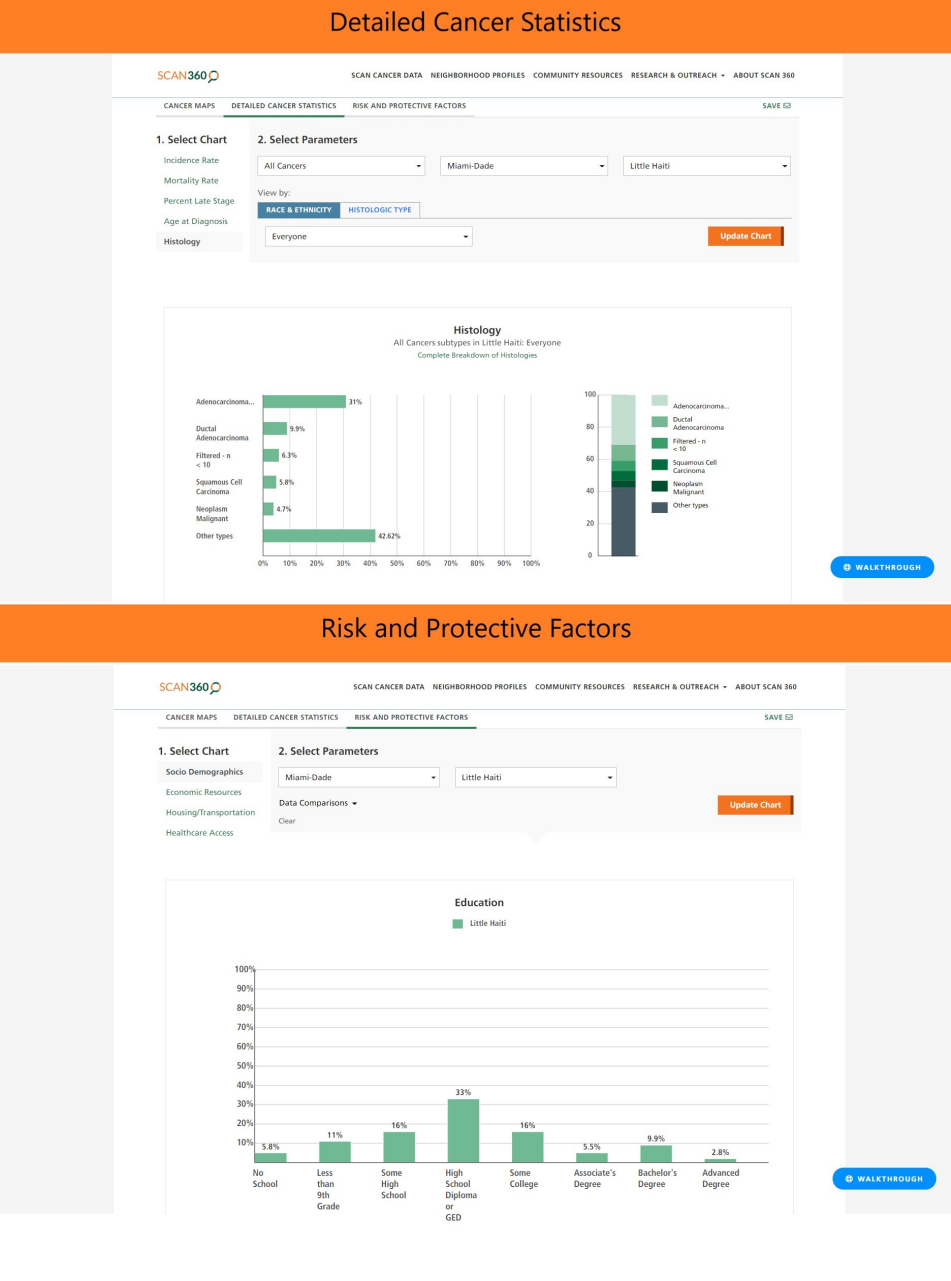
Visualizing exposures and resources using SCAN360.

SCAN360 also provides data about each neighborhood, allowing comparisons of environment (eg, housing stock, rent burden, housing vacancy), sociodemographic composition (eg, race/ethnicity, education, income), and resources (eg, health care access, concentration of poverty) across neighborhoods. If we compare Little Haiti with the City of Miami (the urban center of Miami-Dade County), we see that 70% of residents in Little Haiti have an extreme rent burden, meaning that more than 50% of their income is spent on housing (https://bit.ly/SCANLittleHaitivMiami). Furthermore, we see more housing vacancy and less housing dedicated to “occasional use” (ie, vacation homes). This snapshot of data may reflect secular changes occurring to a greater extent in Little Haiti than in the City of Miami overall. The resources and social support in Little Haiti may be disrupted by sociodemographic and environmental changes in the neighborhood caused by gentrification and displacement, and affect cancer risk, treatment, and survival. In addition to providing information on risk and protective factors, SCAN360 affords the opportunity to delve into detailed cancer statistics, including age at diagnosis, histology, percentage of late-stage diagnosis, and protective resources and detrimental exposures in the community (Figure 2).

In addition, the side-by-side choropleth maps allow comparison not just of different populations; this display allows for the comparison of cancer outcomes with a range of economic, environmental, health behavior, housing, transportation, sociodemographic, and other health characteristics. For instance, zooming in to Golden Glades, North Miami, and West Little River, we can see that many neighborhoods with the highest incidence rates of cervical cancer among Black women are also among the neighborhoods with the lowest levels of median income (https://bit.ly/CervIncome) and health insurance access among women aged 18 to 64 (https://bit.ly/CervicalInsur).

The correlational relationships suggested by the side-by-side maps can spark new research, and we advocate use of the maps primarily for generating hypotheses, to avoid ecological fallacy. Hypothesis generation allows for further innovative investigation through a range of analyses (eg, multilevel, spatial regression). For example, catalyzed by hypothesis generation using basic residential segregation data, ovarian cancer data, and visualizations from SCAN360, researchers conducted an in-depth analysis of the effect of residential segregation on epithelial ovarian cancer survival in Florida by race/ethnicity (13).

## Public Health Implications

Recognizing the multiple levels of interplay in the patterning of health and health inequities, we can use SCAN360 to help identify key geographic areas in which to concentrate resources, build relationships with local community partners, and develop community-tailored policies and programs not only to mitigate cancer burden but also to reduce disparities in cancer. In short, SCAN360 provides a platform and resources to analyze the potential causal interplay between biological, behavioral, social, and structural factors across populations and geographic areas, and, therefore, can help guide cancer control and prevention efforts. For cancer centers in Florida, SCAN360 can help highlight areas of investigation and outreach that are particularly relevant to their catchment areas. Combining cancer registry data with comprehensive data sources allows for directed multilevel research in Florida, a state characterized by cultural heterogeneity. Some areas in North and Central Florida are culturally, demographically, and historically similar to the Deep South, whereas South Florida has multiple enclaves of immigrant residents. The burden of cervical cancer is a particular concern for communities in South Florida, given the concentration of immigrant populations with limited access to human papillomavirus (HPV) vaccination in their countries of birth and their current communities and limited access to methods of secondary prevention (eg, cervical cancer screening, HPV co-testing). Other disease sites or features may be relevant for other cancer centers in the state, allowing each to allocate resources accordingly. In addition, SCAN360 sets the stage for multilevel research. For example, the frailty survival model uses both person-level and neighborhood-level factors to predict a woman’s hazard of death from ovarian cancer (14) using data exported from SCAN360 (an option that should soon easily be accessible on our platform).

All data summarized on SCAN360 are updated annually as soon as the data become available. We plan to make SCAN360 a more comprehensive platform by including diseases and conditions such as HIV and stroke. As the data for these diseases and conditions become available, we will add data on risk and protective factors. The addition of these data may lead to more frequent updates.

We are exploring other ways to enhance the capabilities of the SCAN360 platform, including the addition of basic correlational statistics by geography, bivariate choropleth maps (to display the relationship between 2 categorical variables on the same plot), user-defined neighborhoods/geographic regions, and survival data; expansion into other geographic areas (including New York), and incorporation of social media machine learning into community profiles. As of this writing, in September 2020, SCAN360 shows choropleth maps with color schemes that draw attention to areas with high values. Public health experts know to temper concerns for these highlighted areas if they see wide confidence intervals (which SCAN360 provides on mouse-click or hover-over). We are experimenting with value-suppressing uncertainty palettes, which fade the saturation of the choropleth map toward gray as a function of the width of confidence intervals (15). These maps will help focus attention on problem areas that are relatively certain. Researchers who wish to use the data in SCAN360 can request a data export. We are currently building self-service export tools that will provide analysis-ready data sets to support focused research.

SCAN360 and similar web platforms can provide an accessible way for decision makers, health care professionals, researchers, and community leaders to integrate and visualize comprehensive data across various levels of geography and across groups of race/ethnicity, age, and sex/gender. This increased capacity can promote data-driven decision making and resource allocation, putting surveillance into action.
